# A Novel Anatomic Landmark to Target the Left Ventricle During Chest Compressions in Cardiac Arrest

**DOI:** 10.7759/cureus.13652

**Published:** 2021-03-02

**Authors:** Paul A Olszynski, Rhonda Bryce, Qasim Hussain, Stephanie Dunn, Brandon Blondeau, Paul Atkinson, Robert Woods

**Affiliations:** 1 Emergency Medicine, University of Saskatchewan, Saskatoon, CAN; 2 Clinical Research Support Unit, University of Saskatchewan, Saskatoon, CAN; 3 Emergency Department, Royal University Hospital, Saskatoon, CAN; 4 Faculty of Nursing, University of Regina, Saskatoon, CAN; 5 School of Health Sciences, Saskatchewan Polytechnic, Saskatoon, CAN; 6 Emergency Medicine, Saint John Regional Hospital, Saint John, CAN; 7 Emergency Medicine, Dalhousie University, Halifax, CAN

**Keywords:** ultrasound, anatomy, resuscitation, cardiac arrest, cpr

## Abstract

Background

Resuscitation guidelines recommend that chest compressions be performed over the lower sternum. Current computed tomography and magnetic resonance imaging studies suggest that the current area of compression does not target the left ventricle (LV). Using transthoracic ultrasound, we sought to identify potential anatomic landmarks that would result in compressions over the LV in the majority of our study participants.

Methodology

We recruited 64 healthy men and women (over the age of 40) from the Simulated Patient Program at the University of Saskatchewan. Using ultrasound, we identified the LV and the associated surface anatomy in terms of intercostal space (ICS) and parasternal or mid-clavicular lines. We also collected biometric data including body mass index, chest circumference, and the corresponding inter-nipple line ICS.

Results

The LV was located along the left sternal border in 62 (96.9%) participants. The most frequent LV location was along the left sternal border at the sixth ICS in 26 (40.6%) participants, with 13 (20.3%) at the fifth and 10 (15.6%) participants at the seventh ICS. In two (3.1%) participants, the LV was found along the mid-clavicular zone at the fifth ICS. The area from the fifth to seventh ICS on the left sternal border, typically covered by an adult palm centered at the sixth ICS, overlaid 49 of 64 (76.6%, 95% confidence interval [CI]: 64.3-86.2%) identified LV locations. By comparison, centering the heel of the palm over the inter-nipple line at the left sternal border would cover the LV in 46 (71.9%, 95% CI: 59.2-82.4%) participants.

Conclusions

A novel area of compression over the left sternal border at the inter-nipple line would result in compressions over the LV in nearly three-quarters of our study participants. Future research should investigate whether this proposed area of compression is applicable to a broader population including those with cardiac and thoracic disease.

## Introduction

Despite automated defibrillation and compression-focused resuscitation, out-of-hospital-cardiac-arrest survival remains 10% [1-3]. A mainstay of survival is effective chest compressions. According to the American Heart Association’s cardiopulmonary resuscitation (CPR) guidelines, compressions should be performed over the lower half of the sternum [3]. During CPR, forward blood flow results from a combination of effects on the thoracic cavity and heart, generating flow through what are, respectively, known as the thoracic and cardiac pumps [4-6]. However, emerging research suggests that the current area of compression often results in outflow obstruction that may significantly limit or even compromise compression effect [5,7-10].

A porcine study [11] demonstrated an increase in return of spontaneous circulation when compressions were performed directly over the center of the left ventricle (LV). The optimal compression zone was predetermined using transthoracic echocardiography (TTE) along the left sternal border by identifying the LV in its long axis. In recent years, use of transesophageal echocardiography (TEE) during human cardiac arrest has emerged as a means of guiding resuscitation and improving chest compressions. CPR quality can be improved by moving the area of compression according to visualized LV changes, including greatest LV compression [12,13], alleviation of outflow obstruction [14], and opening of the mitral valve with ventricular filling [15]. Evidence of increased cross-sectional area of the descending aorta immediately proximal to the point of maximal compression suggests that such compressions may have the added benefit of preferential cerebral blood flow [5]. Cardiac arrest guidelines for TEE outline the potential benefit of targeting LV compression to improve perfusion [16-18].

While TTE and TEE can be used to identify the LV and guide compressions, this is not currently a practical solution for out-of-hospital cardiac arrest. Using ultrasound, we sought to localize the LV and then correlate its location to external anatomic landmarks, thus identifying a novel area of compression that would overlie the LV in the majority of our study participants.

## Materials and methods

We obtained approval from the University of Saskatchewan’s Research Ethics Board (Bio #461) to conduct a pair of investigations exploring rapid localization of the LV through either sonographic [19] or anatomic landmark approaches. From January to April of 2019, we recruited a convenience sample of men and women over 40 years of age from the Health Sciences’ Simulated Patient Program (University of Saskatchewan). We collected biometric data including weight, height, and chest circumference at the inter-nipple line. Body mass index (BMI) was calculated as weight (kg)/height^2^ (m) and classified as normal (<25), overweight (25-29.9), or obese (≥30).

**Figure 1:**
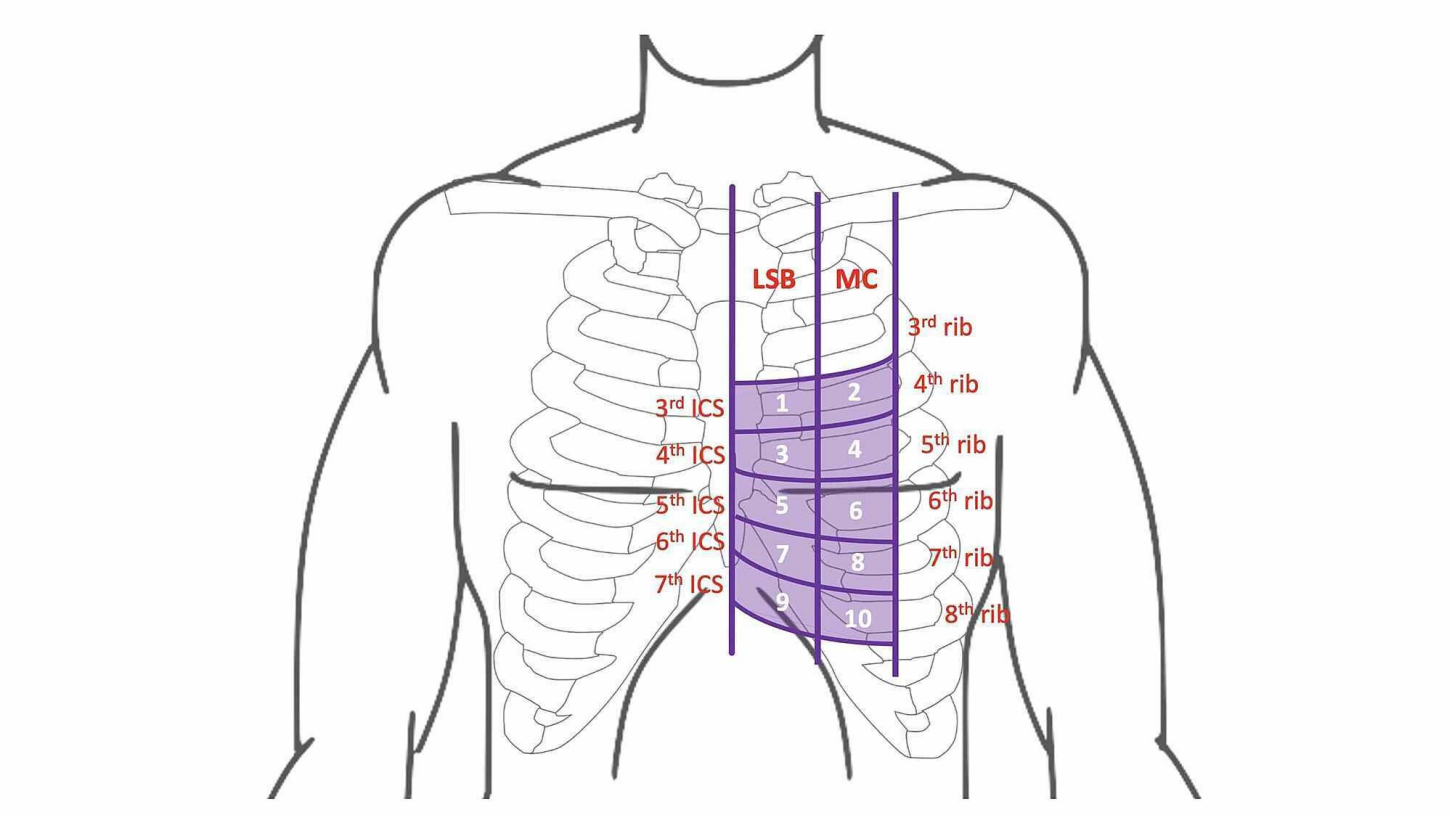
Ten-point interrogation grid. LSB: left sternal border; MC: mid-clavicular; ICS: intercostal space

Participants were placed in a supine position and ultrasound localization of the LV was performed by authors PO and RW using GE Venue 40 machines (General Electric, NY, USA). The ultrasound transducer was oriented along known echocardiographic windows including the parasternal long axis, apical four-chamber, and subcostal views. If a parasternal view of the heart was successfully obtained at the location, the space was deemed to be overlying either the long axis of the LV or aortic root (depending on which was the dominant structure in the view). If an apical four-chamber view was obtained, the space was deemed to be overlying the apex. If a subcostal view was obtained, the space was deemed to be overlying the inferior border of the heart (i.e., the right ventricle). Location of this position was plotted on a grid consisting of ICSs three to seven and two longitudinal zones: the left sternal border and mid-clavicular zones (see Figure 1). The nipple ICS was also documented in this supine position. All relevant images were saved and securely stored for subsequent review, as needed.

For the purpose of this study, we considered successful localization of the LV as an ICS where the long axis of the LV was the dominant structure in view (representing >50% of horizontal axis). Ultrasound was also used to describe the participant’s thorax including a scan for both pleural and pericardial effusions.

In clinical echocardiography, the LV is usually along the left sternal border between the third to fifth ICSs with the patient in left lateral decubitus [20,21]. These landmarks are of limited generalizability to the supine cardiac arrest patient undergoing CPR. Characteristics such as age, BMI, and certain medical conditions further contribute to the variability in terms of LV location [22]. While a detailed parasternal long axis view is not always achievable in the supine patient, it is nonetheless often sufficient to identify gross anatomy such as the LV. The location of the LV via the parasternal window (in terms of ICS and longitudinal line over the chest) represents our theorized optimal area of compression, as supported by porcine findings [11].

The most common means of compression in cardiac arrest is the heel of the human palm, measuring a mean width of 7-8 cm in females and 8-9 cm in males [23,24]. The average fourth to eighth ICSs (defined cranially by the inferior rib margin and caudally by the superior margin of the subsequent rib) show incremental widening from 1.3 cm (±0.3 cm) to 2.1 cm (±0.4 cm), respectively [25]. Combining ICSs and their associated rib widths, the average human palm spans approximately three ICSs. As such, we analyzed our findings in terms of not only the single best ICS overlying the LV but also as a range recognizing the width of the palm, including an ICS above and below the most common location of the LV. We submit that an area of compression where the LV is situated somewhere beneath the heel of the palm may be superior to compressions centered entirely away from the LV.

Descriptive statistics (means with standard deviations, frequencies with proportions) were calculated to characterize the demographic and anatomical features of participants. ICSs of the inter-nipple line and LV location were cross-tabulated to assess consistency; proportions agreeing and their 95% confidence intervals (CIs) were determined. To account for the heel of the hand, agreement between anatomical landmarks and LV location was also assessed allowing for a margin of one ICS above and below the anatomical location. Agreement was assessed for the sample as a whole and by subgroups of sex and BMI. Exact CIs were determined for sex-specific and BMI category-specific proportions due to the relatively small number of participants in the subcategories. Proportions were compared between subgroups using the chi-square test. IBM SPSS Statistics for Windows, version 27 (IBM Corp, Armonk, NY, USA) was used for analysis.

## Results

Demographic and anatomical details for participants are outlined in Table 1. The LV was found along the left sternal border in 62 (96.9%) participants. The most frequent location of the LV was the sixth ICS along the left sternal border in 26 (40.6%) individuals, with 13 (20.3%) at the fith and 10 (15.6%) at the seventh ICS. The area from the fifth to seventh ICS on the left sternal border captured 49 of 64 or 76.6% (95% CI: 64.3-86.2%) of the identified LV locations.

**Table 1:** Demographic and anatomical characteristics of participants. BMI = body mass index; SD = standard deviation; y: years

Characteristic		Value	
Age, y, mean (SD)		64.0	(10.5)
Sex, n (%)	Male	27	(42.2)
	Female	37	(57.8)
BMI, mean (SD)		29.0	(6.4)
BMI, n (%)	Normal (<25)	17	(26.6)
	Overweight (25–29.9)	23	(35.9)
	Obese (≥30)	24	(37.5)
Chest circumference, cm, mean (SD)	Overall	98.4	(11.4)
	Males	99.9	(9.2)
	Females	97.3	(12.8)
Inter-nipple line intercostal space	4^th^	8	(12.5)
	5^th^	19	(29.7)
	6^th^	21	(32.8)
	7^th^	14	(21.9)
	8^th^	2	(3.1)

The ICS of the inter-nipple line and LV were consistent among 21 (32.8%, 95% CI: 21.6-45.7%) participants, all with the LV at the left sternal border. The region at the left sternal border covering one ICS above the inter-nipple line to one ICS below it overlaid the LV in 46 (71.9%, 95% CI: 59.2-82.4%) participants. The degree of agreement between the level of the inter-nipple line and the LV are presented in Table 2.

**Table 2:** Study findings for the frequency of LV position and ICSs. LV = left ventricle; ICS = intercostal space; LSB = left sternal border; MCL = mid-clavicular line

Location by echocardiography		Corresponding inter-nipple line ICS, n						
	n (%)	4^th^		5^th^	6^th^		7^th^	8^th^
ICS 3^rd^, LSB	4 (6.3)	1		1	0		2	0
4^th^, LSB	9 (14.1)	1		4	3		1	0
5^th^, LSB	13 (20.3)	1		6	3		1	2
5^th^, MCL	2 (3.1)	1		0	1		0	0
6^th^, LSB	26 (40.6)	4		6	10		6	0
7^th^, LSB	10 (15.6)	0		2	4		4	0
Total	64 (100%)	8		19	21		14	2

Localization of the LV location by anatomic landmarks is described in Table 3. Evaluating identification of the LV location by the inter-nipple line when stratified by sex or BMI, 15 of 37 (40.5%, 95% CI: 24.8-57.9%) females and six of 27 (22.2%, 95% CI: 8.6-42.3%) males had exact matches between inter-nipple line ICS and LV ICS (p = 0.12); 27 (73.0%, 95% CI: 55.9-86.2%) females and 19 (70.4%, 95% CI: 49.8-86.2%) males were shown to have the LV within one ICS of the inter-nipple line at the left sternal border (p = 0.82). Among BMI subgroups, eight of 17 (47.1%, 95% CI: 23.0-72.2%) normal BMI, four of 23 (17.4%, 95% CI: 5.0-38.8%) overweight, and nine of 24 (37.5%, 95% CI: 18.8-59.4%) obese participants demonstrated an exact match between the two locations (p = 0.12); 13 of 17 (76.5%, 95% CI: 50.1-93.2%), 17 of 23 (73.9%, 95% CI: 51.6-89.8%), and 16 of 24 (66.7%, 95% CI: 44.7-84.4%) had their LV within one ICS above or below the nipple line at the left sternal border (p = 0.76).

**Table 3:** Study findings describing LV position in relation to anatomic landmarks. LV = left ventricle; ICS = intercostal space; LSB = left sternal border; CI = confidence interval

LV location	n (%)	95% CI for proportion
LV found at 6^th^ ICS, LSB, n (%)		
Yes	26 (40.6)	28.5, 53.6
No	38 (59.4)	
LV found within one ICS of the 6^th ^ICS (i.e., under palm coverage), LSB, n (%)		
Yes	49 (76.6)	64.3, 86.2
No	15 (23.4)	
LV found at nipple line, LSB, n (%)		
Yes	21 (32.8)	21.6, 45.7
No	43 (67.2)	
LV found within one ICS of nipple line (i.e., under palm coverage), LSB, n (%)		
Yes	46 (71.9)	59.2, 82.4
No	18 (28.1)	

## Discussion

Our findings demonstrate that the LV is found to the left of the sternal border in a majority of supine adult participants. An area of compression over the left sternal border at either the sixth ICS or the inter-nipple line would result in compressions over the LV in the majority of our study participants. We report both of these anatomic locations because, despite evidence of improved LV localization at the sixth ICS, palpation of ICSs is challenging and has been found for chest tube insertion [26]. In contrast, the intersection of the left sternal border at the inter-nipple line presents a readily identifiable anatomic landmark for first responders. Differences between sex and BMI groups, though not statistically significant, warrant further consideration and investigation as obesity may limit generalizability.

Multiple radiological studies evaluating the location of critical cardiac structures have been published. In computed tomography (CT) and magnetic resonance imaging (MRI) studies, it has been demonstrated that the lower half of the sternum mostly compresses structures other than the LV including the ascending aorta, left atrium, right ventricle, aortic root, and proximal descending aorta [5,7-9].

A case series of five patients with TTE-guided LV compressions showed promising improvements in oxygen saturation and end-tidal CO_2_, but no improvement in survival. This may be attributed to the duration of standard compressions prior to LV localization and subsequent targeted LV compression [27]. The authors proposed integrating TTE at the start of CPR to optimize LV compression from the onset of resuscitation efforts. Similarly, TEE-guided compressions have not been shown to improve survival, possibly because this modality is often only implemented well into cardiac arrest care and the effects of targeted LV compression are blunted. Proposals to move the recommended hand position for chest compressions inferiorly and/or laterally to the left of the sternum have been previously made but met with reluctance due to concerns about possible impairment of the thoracic pump effect, as well as concern for injury to intra-abdominal organs [8,10,21].

Our study has several limitations. First, while we propose a novel area of compression overlying the LV, we present no data on the actual effects such a move would have on human patients during cardiac arrest in terms of hemodynamics, trauma to internal organs, survival, or other clinical outcomes. Our sample size was relatively small, and even though we did not screen our participants, the majority were generally healthy. Patients who suffer cardiac arrest likely have a higher prevalence of cardiac and thoracic pathology, most importantly cardiac dilation, which may result in lateral displacement of the LV and its axis. We used ultrasound to localize the heart as opposed to CT or MRI, and we did not perform blinded comparisons of location to ensure inter-rater reliability. While cardiac ultrasound is a user-dependent skill, both authors performing the scans have completed training in focused cardiac ultrasound consistent with the guidelines set forth by the Canadian Association of Emergency Physicians [28].

## Conclusions

Utilizing ultrasound to map the position of the LV to the surface anatomy of our adult participants, we were able to identify an area of compression that may result in LV compression in most patients. Performing chest compressions over the left sternal border at the intersection of the inter-nipple line should be feasible for first responders and could optimize chest compression in cardiac arrest in a majority of patients. Further investigation is warranted to determine if the proposed area of LV compression is valid in a larger cohort of patients including those with cardiac and thoracic disease.
